# Lung Metastases: Current Surgical Indications and New Perspectives

**DOI:** 10.3389/fsurg.2022.884915

**Published:** 2022-04-29

**Authors:** Giuseppe Mangiameli, Ugo Cioffi, Marco Alloisio, Alberto Testori

**Affiliations:** ^1^Division of Thoracic Surgery, IRCCS Humanitas Research Hospital, Milan, Italy; ^2^Department of Biomedical Sciences, Humanitas University, Milan, Italy; ^3^Department of Surgery, University of Milan, Milan, Italy

**Keywords:** lung metastases, pulmonary metastasectomy, thoracic surgery, wedge resections, indications, secondary lung cancer

## Abstract

Pulmonary metastasectomy is an established treatment that can provide improved long- term survival for patients with metastatic tumor(s) in the lung. In this mini-review, we discuss the state of the art of thoracic surgery in surgical management of lung metastases which actually occurs for a large part of surgical activity in thoracic surgery department. We describe the principles of surgical therapy that have been defined across the time, and that should remain the milestones of lung metastases treatment: a radical surgery and an adequate lymphadenectomy. We then focus on current surgical indications and report the oncological results according to the surgical approach (open vs. mini-invasive), the histological type and number of lung metastases, and in case of re-metastasectomy. Finally, we conclude with a brief overview about the future perspectives in thoracic surgery in treatment of lung metastases.

## Introduction

Approximately 30% of patients with a malignant disease will develop pulmonary metastases (1). The most common primary solid tumor cause of pulmonary metastases is a carcinoma of the colon and rectum, kidney, breast, prostate, and oropharynx. Additionally, tumors that preferentially metastasize to the lungs are also chorionic carcinoma, osteosarcoma, soft tissue sarcoma, testicular tumors, Ewing sarcoma, and thyroid carcinoma (2).

In 1997, a long-term prognostic analysis on 5,206 lung metastasectomies showed that survival after complete resection was 36, 26, and 22% at 5, 10, and 15 years, respectively, with a median survival of 35 months. Based on these findings, pulmonary metastasectomy (PM) has been commonly introduced in thoracic surgery as therapeutic option that can provide improved long- term survival for patients with lung metastases (3, 4).

Thus, today, PM represents a very significant portion of the activity of a thoracic surgery department. It is not surprising that according to a recent report by the Committee for Scientific Affairs of the Japanese Association for Thoracic Surgery, PM accounted for as many as 10.2 % of all entry cases of general thoracic surgery, and its use is increasing year by year (5).

In this mini-review, we discuss the state of the art of thoracic surgery in the management of lung metastases describing the principles of surgical therapy that have been defined over time, and which should remain the milestones of lung metastases treatment: a radical surgery with free margins considering a lymphadenectomy. We will therefore focus on the surgical approach (open vs. mini-invasive) and on the different oncological results according to the histological type, number of lung metastases, and after a re-do PM. Finally, we will conclude with a brief overview of the future perspectives in thoracic surgery in the treatment of lung metastases.

## Principles of PM

From the first described PM, several cases have been reported in literature over time (6). PM was initially evaluated in patients with tumors of various origins, and surgical criteria have been proposed based on retrospectives case series data (7).

Recently, the general criteria that should always be observed before referring patients to metastasectomy have been resumed by the Society of Thoracic Surgeons (STS) Work Force of Evidence Based Surgery (8). The most important are (I) primary cancer control, (II) absence of other extra-thoracic metastases, and (III) complete metastasis resection (8).

Although substantial agreement exists in performing surgery limited to these criteria, there are no official guidelines defining the optimal surgical approach and type of resection, nor whether perioperative lymph node (LN) evaluation should be performed for these patients (9).

The main goal of PM is to achieve a complete resection of the metastases while preserving as much pulmonary parenchyma as possible. The goal of radical resection is generally obtained through wedge resections (WRs) or surgical excision by electrocautery or laser ablation for peripheral lesions. Conversely, anatomical resection such as segmentectomy, lobectomy, or pneumonectomy may be necessary to ensure radical resection of central lesions.

According to the data published by the International Registry of Lung Metastases in 1997, the most common procedure performed was WR in 67% of cases, followed by segmentectomy in 9%, lobectomy in 21%, and pneumonectomy in 3% (4). These data are comparable to those reported by a recent analysis of current surgical practice outcomes of PM, based on the European Society of Thoracic Surgeons database according to which WR was the most common performed procedure (61%) followed by an anatomical resection in 39% of cases with lobectomy, segmentectomy, bilobectomy, and pneumonectomy managed, respectively, in 39, 26, 1, and 1% (9).

These data confirm that the trend in the frequency of surgery does not appear to have changed much over time with the majority of lung metastatic lesions located at the periphery of the lung and easily accessible to WR. On the other hand, pneumonectomy to accomplish PM is actually not recommended except in carefully selected patients undergoing multidisciplinary team management. It is not a coincidence if the rate of performed pneumonectomy comes from 3 (4) to 1% in the last decades (9). Probably, this reduction is the confirmation that it is generally agreed among surgeons that pneumonectomy should only be kept as the last resort for metastasectomy in highly selected patients and for very clear surgical and medical indications considering that it massively impairs respiratory functions (9).

## Thoracotomy vs. Mini-Invasive Surgery

Traditionally, thoracotomy with manual palpation has been proposed as the standard surgical approach for performing PM. The main advantage of thoracotomy has always been the possibility of performing a bimanual palpation avoiding missing nodules that would have remained undetected during preoperative radiological examinations. Furthermore, in recent years, video-assisted thoracic surgery (VATS) has been progressively and largely adopted for performing PM procedures even if this utility for treating pulmonary metastases remains unclear. The main problem remains that finger palpation through port sites or utility incisions as well as indirect palpation of the lung using instruments for pulmonary metastasis is sometimes difficult or impossible during VATS. Several studies report that small or minute non-imaged lung nodules can be missed during surgery (10, 11).

An interesting prospective observer-blinded study reports as a substantial number of additional nodules were detected during thoracotomy performed immediately after VATS, and many of these nodules were malignant and would have been lost if VATS had been used exclusively. The authors conclude that VATS was inadequate if the intention is to resect all pulmonary metastases during surgery (12). On the other hand, several authors reported that disease free survival (DFS) did not appear to be affected by the approach, at least for colorectal metastases (13), and others that overall survival and recurrence survival did not differ between vats and open PM independently of the type of metastatic primary tumor (14–18).

In a recent mini-review, it was confirmed that all thoracoscopic resections compared to open surgery were associated with better short-term outcomes, shorter hospital stays, chest drainage duration, and fewer perioperative complications in two studies. Furthermore, no survival differences were identified with either approach (17).

Another recognized advantage of VATS is the reduced invasiveness avoiding the reduction of pleural adhesion in treating patients that probably will be submitted to surgery many times. Furthermore, the possibility to perform a hybrid metastasectomy technique involving a combination of VATS and mini-thoracotomy or hand-assisted thoracoscopic surgery has been developed to overcome the disadvantages of VATS PM (19). Thus, it is not surprising if actually in Japan more than 70 % of PM procedures are performed using VATS (5). Similarly in Europe, the rate of VATS procedures significantly increased from 15% in 2007 to 58% in 2018 as reported by ESTS report (9).

In conclusion, the recommendation of expert consensus document on PM is that in oncological and medically appropriate patients, PM can be considered with a preference for mini-invasive surgery owing to the shortened postoperative recovery and reduced effect on short-term quality of life. If the goals of R0 and pulmonary parenchymal sparing are not achievable with mini-invasive surgery but lend themselves to open approaches (thoracotomy, sternotomy, or clamshell), open techniques are appropriate (8).

## Surgical Margin

Staplers, electric scissors, laser scissors, and coagulation instruments are common devices used in performing PM (20). Regardless of the surgical device adopted, postoperative local recurrence at the surgical margin still remains an important problem ranging from 4 to 31% after PM procedures (21–25).

Surgical margin distance is recognized as a critical point of WR that is the most common procedure performed in treating pulmonary metastases. Actually, in clinical practice, the tumor-free surgical margin is checked macroscopically and, if necessary, by histological examinations of frozen sections. Although the surgical margin appears macroscopically to be sufficient, about 10% of the resections may be microscopically incomplete (3). To prevent local recurrence, Rusch advised removing a cone-shaped pulmonary parenchyma wedge circumferentially around the nodule and to take a 0.5–1.0 cm margin of normal lung tissue with it in all directions (3). However, significant increases in local recurrence were found in cases of resected pulmonary metastases with a surgical margin distance of less than 7 mm. Satellite cancer cells, a potential source for local recurrence, were identified in 99.7% of nodules within 7.4 mm of the tumor (26). Therefore, several authors suggest avoiding such failure, a WR with a sufficient margin of 10 or 20 mm if possible (26, 27).

Other factors influencing surgical margins and a possible local recurrence are the size and the tumor location. A recent study demonstrated that larger metastatic tumors had a higher risk of local recurrence (28). Thus, depending on the tumor size, the safety margins may need to be increased. For these reasons increasing importance is given to the new prognostic factor as tumor/margin ratio (28). Tumor location also plays an important role in preventing recurrence at the surgical margin considering that the achievement of a sufficient surgical margin depends on tumor site. In case of tumors located in the edge of the lung, a sufficient surgical margin could be easily obtained. Conversely, in case of tumors located in the large ovoid face, e.g., basal segment, a sufficient surgical margin could not be obtained (29). Shiono et al. suggested WR for peripheral lung nodules and segmentectomy for more central lesions (30). Segmentectomy is known to achieve a larger surgical margin than WR. Lower surgical margin recurrence rates have been reported with segmentectomy (2%) compared to WR (7.3%) for colorectal cancer lung metastases (31). Table 1 resumes all evidences reported about surgical margin.

**Table 1:** Surgical margin and suggested procedure.

**Author**	**Years**	**Suggested surgical margin or procedure**	**Rationale**
Rusch (3)	1995	5 to 10 mm	–
Welter et al. (26)	2011	7 mm	Satellite cancer cells, identified in 99.7% of nodules within 7.4 mm of the tumor
Chung et al. (28)	2019	Depending of tumor size (tumor/margin ratio) it should be increased	Larger metastatic tumors had a higher risk of local recurrence
Shiono et al. (30)	2021	Segmentectomy or WR according to nodule site	Segmentectomy in case of central location

## Lymphadenectomy

In patients with lung metastases from an extrathoracic solid organ, intrathoracic LN involvement is a poor prognostic indicator (32, 33).

Historically, thoracic surgeons rarely perform mediastinal LN dissection in the setting of metastatic disease. However, this attitude has changed across the time with an LN assessment which increased from 4.6% in 1997 (4) to 58% in 2021 (9).

Although current evidence suggests that intrathoracic LN status is an important predictor in PM, there are no randomized data that respond to mediastinal lymphadenectomy having a therapeutic effect. However, in a recent cross-sectional survey, both preoperative tissue assessment of radiologically suspicious LNs and intraoperative assessment are “recommended” by the expert panel (34). Furthermore, the recommendation of expert consensus on PM is that LN sampling/dissection concomitant with PM should be considered, because pulmonary metastasis accompanied by mediastinal LN metastasis predicts poor survival (8).

## Surgery According to the Histological Type

Since each histological type behaves differently, it is reasonable to assume that the efficacy and role of surgery depend on the primary tumor histology. Regardless of histological type, several prognostic factors have been described as predictors of a worse prognosis such as incomplete resection, number and the size of resected tumor, LN metastases, and a short disease-free interval (DFI) (35). Conversely, other predictors are specific to certain histological type and are reported in the following.

PM is considered a potentially curative treatment for patients affected by metastatic sarcoma with a reported 5-year overall survival rates after resection ranging from 15 to 50.9% (36–39). The most common negative prognostic factor reported are high-risk histology, grade 3 (G3) sarcoma, and the bilaterality of lung metastases (38).

Colorectal cancer is the most common primary tumor in patients who undergo PM; several studies confirm that colorectal carcinoma is a favorable histological subtype for metastasectomy (40, 41) reporting excellent 5-year survival rates up to 68% (42). Preoperative serum carcinoembryonic antigen (CEA) level, patient >70 years old, the extrathoracic metastatic lesions treated curatively before PM resection, and rectal location are the most common poor prognostic factors specific for this histology (43–46).

Renal cell carcinoma is the second-most common primary tumor in patients undergoing PM but most recent studies shown as this histology is exclusively related to the abovementioned common prognostic factors (47).

Less favorable evidence is reported concerning resected metastases of head and neck carcinomas (48) with reported 5-year overall survival rates ranging from 20.9 to 59.4% (49).

Adenoid cystic carcinomas have been associated with a better prognosis compared to head and neck squamous cell carcinoma (50). Furthermore, old age and the occurrence of local recurrence before lung metastases have been reported as factors associated to a worse prognosis and poor overall survival (51).

In the field of gynecological cancer, 5- and 10-year survival rates of 40.9 and 31.4%, after PM have been reported (52). A factor predictive of poor survival is cervix primary lesion. Finally, with regard to breast cancer, the level of evidence for a curative approach is low and a less favoritism to PM is most likely due to the improvement of systemic therapies effective in prolonging life in the disease (53).

On Table 2 are resumed specific survival predictors and reporting 5 years overall survival according to different histological type.

**Table 2:** Specific predictors and reported 5 years OS survival according to different histological type.

**Histology**	**Specific predictors**	**5 years OS**
Sarcoma (36–38)	- High-risk histology	From 15 to 50.9%
	- Grade 3 (G3) sarcoma	
	- Bilaterality of lung metastases	
Colorectal cancer (43–46)	- Preoperative CEA level	Up to 68%
	- Patient >70 years old,	
	- Extra-thoracic metastatic lesions treated curatively before PM resection	
	- Rectal location	
Renal cell carcinoma (47)	- None	75%
Head and neck carcinomas (48–51)	- Histology	From 20.9 to 59.4%
	- Old age	
	- Occurrence local recurrence before PM	
Gynecological cancer (52)	- Cervix primary lesion predictive of poor survival	40.9%

## Surgery Based on the Number of Lung Metastases

The number of metastatic lesions discovered before or at operation is a well-studied and important prognostic variable (54). Most authors would agree that a larger number of lesions (≥3) are associated with a poor prognosis (4) but the cutoff value for denying PM for patients with multiple lung metastases (LM) is undetermined. Interestingly Girard et al. reported that the prognostic value of the number of metastases is greater for patients with a carcinoma than for those with sarcoma (55).

## Re-do Surgery

Usually, repeated PM for metachronous pulmonary metastases is mainly performed in patients with colorectal cancer, renal cell cancer (RCC), or bone/soft tissue sarcoma. Resection of recurrent metastases should be considered within a multidisciplinary team and carefully individualized to define whether repeat resection is indicated. The surgical indications for repeated PM do not differ from those for the initial operation, but preoperative evaluations should be performed more carefully to ensure complete a surgical resection while maintaining physical function (56–58). Several factors such as DFI, overall prognosis, and expected benefit of medical treatment should be considered in decision-making. Usually, a longer time interval between the first metastasectomy and the appearance of recurring metastases appears to be prognostically more favorable (4, 59). Thus, if the surgical indication for metastatic lung tumor is satisfied and the prognostic factors are met, re-surgery should be actively considered with reasonable expectations of long-term survival even now that the drug therapy is advanced (60–62).

## Innovation and Future Perspective in Thoracic Surgery

The introduction of the radial stapler, the use of intraoperative near infrated (NIR) imaging, and laser-assisted surgery (LAS) represent some of the innovations recently introduced in the field of PM. In the same way, the availability of new drugs and experimental surgical techniques contribute to this innovation process.

Up to date, few publications describe the use of radial stapler in thoracic surgery. Compared to a linear stapler-only option, the radial stapler may help thoracic surgeons preserve lung parenchyma during WRs while maintaining adequate margins (63).

Fluorescence is a new technology which has spread concurrently with mini-invasive surgery. In recent years, new optical system has been created and commonly adopted during mini-invasive surgery (64). Recently, the use of NIR intraoperative imaging with indocyanine green (5 mg/kg and 24 hours before surgery) has been reported as useful tool in localizing the known sarcoma pulmonary metastases and identifying otherwise occult lesions (65). This approach has been also described in performing thoracoscopic PM of HCC metastases by simplifying tumor locations and ensuring resection margins (66).

Laser-assisted surgery is a recent innovation that has been advocated especially in patients with multiple lung metastases. LAS have the advantages to allow a complete resection of a significantly higher number of metastases compared to stapling resections and to be a tissue-saving technique which allows repeated resections in case of recurrence (67, 68).

Recently, experimental surgical techniques such as isolated lung perfusion with melphalan have also shown promising results in phase I and II studies in patients affected by resectable pulmonary metastases of the colorectal carcinoma, osteosarcoma, and soft tissue sarcoma (42, 69).

## Conclusion

Pulmonary metastasectomy is a well-recognized and established treatment that can provide improved long- term survival for patients with metastatic tumor(s) in the lung. WR is the most common procedure performed allowing to satisfy the main goal of PM that is to achieve a complete resection of the metastases while preserving as much pulmonary parenchyma as possible. Instead, an anatomical resection such as segmentectomy, lobectomy, or pneumonectomy may be necessary to ensure radical resection of central lesions. Actually, the major part of PM is performed by mini-invasive surgery allowing several advantages compared to open lobectomy (less pain, shorted postoperative recovery and better quality of life) maintaining R0 resection. It should be associated to an adequate intraoperative LN sampling considering the known importance of LN involvement in determining a worse prognosis. Incomplete resection, the number and the size of resected tumor, the presence of LN metastases, and a short DFI are all prognostic factors of worse survival independently of histological type.

Several innovations have been introduced and probably will change the landscape and treatment guidelines for patients with metastatic lung disease.

## Author Contributions

All authors listed have made a substantial, direct, and intellectual contribution to the work and approved it for publication.

## Conflict of Interest

The authors declare that the research was conducted in the absence of any commercial or financial relationships that could be construed as a potential conflict of interest. The reviewer FP declared a shared affiliation with the author UC to the handling editor at the time of review.

## Publisher's Note

All claims expressed in this article are solely those of the authors and do not necessarily represent those of their affiliated organizations, or those of the publisher, the editors and the reviewers. Any product that may be evaluated in this article, or claim that may be made by its manufacturer, is not guaranteed or endorsed by the publisher.
